# Diagnostic utility of high-resolution magnetic resonance vessel wall imaging for identifying culprit plaques in intracranial atherosclerotic disease

**DOI:** 10.3389/fneur.2026.1677672

**Published:** 2026-03-04

**Authors:** Qing Li, Kunheng Fan, Feifeng Liu, Shuguang Chu, Jianhua Zhang, Changlong Hou

**Affiliations:** 1 Department of Radiology, Shanghai East Hospital, Tongji University School of Medicine, Shanghai, China; 2 Department of Neurology, Shanghai East Hospital, Tongji University School of Medicine, Shanghai, China

**Keywords:** acute ischemic stroke, culprit plaque, high-resolution magnetic resonance vessel wall imaging, intracranial atherosclerosis, intra plaque hemorrhage, plaque enhancement, T1WI hyperintensity

## Abstract

**Background:**

Gadolinium enhancement and T1-weighted hyperintensity in intracranial atherosclerotic plaques are recognized markers of plaque instability. This study aimed to evaluate the utility of plaque enhancement grading and T1-weighted imaging (T1WI) hyperintensity in identifying culprit plaques in patients with intracranial atherosclerosis.

**Methods:**

A retrospective analysis was conducted on high-resolution magnetic resonance vessel wall imaging (HRMR-VWI) data from patients with symptomatic intracranial atherosclerosis. Detected plaques were categorized as culprit, possible culprit, or non-culprit plaques based on clinical and imaging criteria. Plaque enhancement and T1WI hyperintensity were qualitatively assessed. Associations between these imaging features and culprit plaques were examined using logistic regression analysis.

**Results:**

The study included 69 patients, comprising 60 with acute ischemic stroke (≤ 1 month after the ischemic event), 7 with chronic ischemic stroke (> 1 month), and 2 with transient ischemic attack. A total of 474 intracranial atherosclerotic plaques were identified: 72 (15.19%) were categorized as culprit, 132 (27.85%) as possible culprit, and 270 (56.96%) as non-culprit plaques. Multivariate logistic regression analysis demonstrated that grade II enhancement (*p* < 0.001) and T1WI hyperintensity (*p* = 0.018) were independently associated with culprit plaques.

**Conclusion:**

Grade II enhancement and T1WI hyperintensity are independently associated with culprit intracranial plaques and may serve as imaging biomarkers of plaque instability, offering valuable insights into stroke risk.

## 1 Introduction

Acute ischemic stroke remains a leading cause of mortality and long-term disability globally. Intracranial atherosclerosis (ICAS) constitutes a major etiological factor in ischemic stroke, particularly among Asian populations, where ICAS-related strokes account for approximately 9 to 33% of all ischemic strokes and are associated with a high risk of recurrence (1, 2). Historically, severe arterial stenosis has been regarded as a primary indicator of cerebral ischemia. However, an increasing body of evidence indicates that the severity of luminal stenosis does not consistently correlate with the occurrence of ischemic stroke (3–5). Studies have reported no statistically significant differences in the degree of stenosis between symptomatic and asymptomatic individuals with moderate to severe middle cerebral artery (MCA) stenosis (6, 7). Therefore, it is now widely accepted that evaluating ICAS should extend beyond the assessment of luminal narrowing to include vessel wall characteristics (8–10).

High-resolution magnetic resonance vessel wall imaging (HRMR-VWI) has increasingly been utilized to delineate the compositional features of intracranial arterial plaques, such as calcification, fibrous caps, lipid cores, and intraplaque hemorrhage (IPH). Moreover, HRMR-VWI findings have demonstrated a high degree of consistency with histopathological analyses, making it a valuable tool for detecting intracranial atherosclerotic plaques and quantifying plaque burden (11–14). Therefore, the present study aims to evaluate whether contrast enhancement grading and T1-weighted imaging (T1WI) hyperintensity of intracranial plaques are useful in identifying culprit plaques and enhancing risk stratification in patients with ICAS.

## 2 Materials and methods

### 2.1 Study population

This retrospective study included 80 patients who presented with symptoms of ischemic stroke and received treatment at Shanghai East Hospital (South Division) between April 2018 and July 2024.

The inclusion criteria were as follows: (1) age > 18 years; (2) intracranial arterial stenosis confirmed by digital subtraction angiography (DSA), involving one or more of the following segments: C6–C7 segment of the internal carotid artery, A1–A2 segments of the anterior cerebral artery, M1–M2 segments of the MCA, V4 segment of the vertebral artery, basilar artery, or P1–P2 segments of the posterior cerebral artery; (3) All patients with HRMR-VWI performed using the same MRI scanner and at least one identifiable intracranial atherosclerotic plaque; (4) acute cerebral infarction confirmed by diffusion-weighted imaging (DWI), or chronic infarction identified on both T2 fluid-attenuated inversion recovery (T2 FLAIR) and DWI sequences.

The exclusion criteria were as follows: (1) evidence of non-atherosclerotic intracranial vasculopathies such as moyamoya disease, reversible cerebral vasoconstriction syndrome (RCVS), arterial dissection, or vasculitis; (2) stenosis of ≥ 50% in the ipsilateral extracranial carotid artery; (3) presence of cardioembolic stroke risk factors, such as recent myocardial infarction within the preceding 3 weeks, atrial fibrillation, or the presence of cardiac or valvular thrombus detected by echocardiography or other imaging modalities; and (4) poor image quality deemed inadequate for diagnostic or analytical purposes. Clinical data collected for each patient included sex, age, hypertension, diabetes mellitus, history of stroke, coronary artery disease, hyperlipidemia, as well as smoking and alcohol consumption history. Imaging for acute stroke patients should be completed within 24 h of symptom onset, for chronic stroke patients 1 month after symptom onset, and for transient ischemic attack patients within 24 to 48 h of symptom onset.

### 2.2 Magnetic resonance imaging (MRI)

All MRI scans were performed using a 3.0 T uMR770 MRI scanner (United Imaging Healthcare Co., Ltd., Shanghai, China) equipped with a 24-channel head-and-neck coil. The imaging protocol included conventional brain MRI, three-dimensional time-of-flight magnetic resonance angiography (3D-TOF MRA), and HRMR-VWI. The HRMR-VWI protocol comprised three sequences: 3D-MATRIX T1WI, fat-suppressed 3D-MATRIX T1WI (T1WI FS), and contrast-enhanced 3D-MATRIX T1WI (CE-3D-MATRIX T1WI). The contrast-enhanced images were acquired 40 s following intravenous administration of the agent. Scan parameters for the 3D-MATRIX T1WI were as follows: repetition time (TR), 821 ms; echo time (TE), 14.76 ms; inversion time (TI), 588 ms; echo train length (ETL), 42; number of excitations (NEX), 1.5; slice thickness, 0.70 mm; and field of view (FOV), 180 × 180 mm. The contrast agent used was gadopentetate dimeglumine (BAYER, batch number KTOLLJF), administered intravenously at a dose of 0.2 mL/kg, with an injection rate of 2 mL/s, the dose of which is the routinely recommended dose for enhanced MRI of the brain.

### 2.3 Image analysis

#### 2.3.1 Plaque identification

HRMR-VWI scans were reviewed on an offline workstation (IMPAX). Atherosclerotic plaques were defined as eccentric thickening of the intracranial arterial wall, with or without associated luminal stenosis, as visualized on HRMR-VWI. Image quality was assessed based on the following criteria: Grade 0, the arterial wall and lumen were not visualized; Grade I, partial visualization of the arterial wall or lumen; Grade II, clear delineation of both the arterial wall and lumen, with well-defined wall structures (15). Only images rated as Grade II were included in this study. Due to the limited diameter of intracranial vessels, the analysis focused primarily on plaques located in large proximal intracranial arteries, including the C6 and C7 segments of the internal carotid artery, M1 and M2 segments of the MCA, A1 and A2 segments of the anterior cerebral artery, P1 and P2 segments of the posterior cerebral artery, the basilar artery, and the V4 segment of the vertebral artery. All identifiable plaques within these vascular segments were recorded.

Culprit plaques were defined according to the following criteria: (1) the only lesion present within the vascular territory corresponding to the infarct; or (2) the presence of multiple plaques within the same territory, the lesion located at the site of maximal luminal narrowing. When multiple plaques exist in the same vascular region, a plaque was considered probably a culprit plaque when it was not the most stenotic lesion within the same vascular territory of the stroke. Lesions located outside the infarct-related vascular territory were classified as non-culprit plaques (16). Plaque identification was independently performed by two neuroradiologists with 10 and 3 years of experience, respectively. Any discrepancies in the classification of culprit plaques were resolved through consensus discussion, supervised by a senior neuroradiologist with 14 years of experience in neuroradiology.

#### 2.3.2 Plaque characteristic assessment

**Figure 1:**
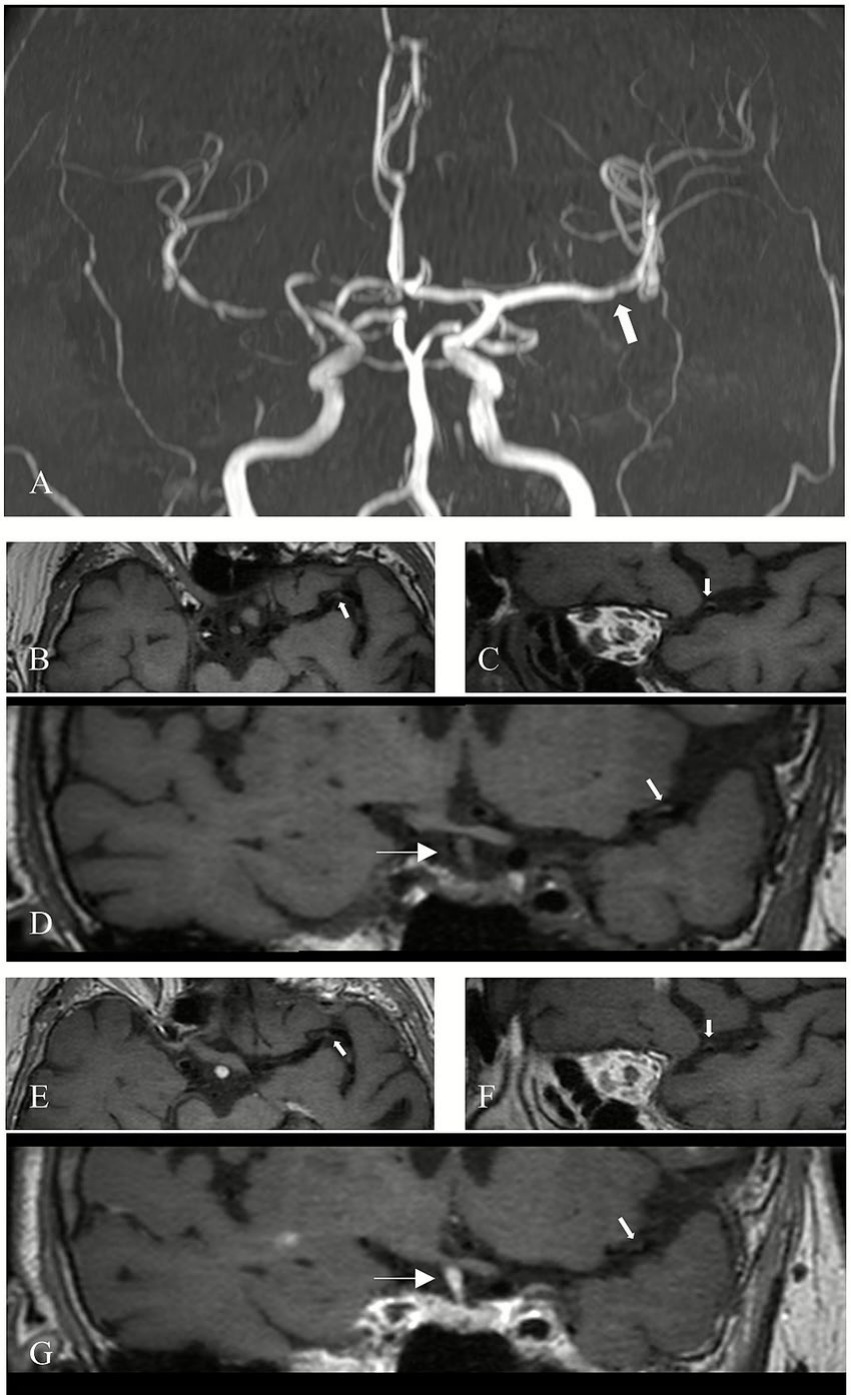
Grade 0 enhancement of a non-culprit plaque. **(A)** MRA demonstrates mild stenosis of the left middle cerebral artery (M1 segment) in a patient (arrow). **(B)** Axial 3D-MATRIX T1WI shows eccentric arterial wall thickening (arrow). **(C)** Sagittal reconstruction perpendicular to the direction of blood flow shows the eccentric plaque (arrow). **(D)** Coronal image shows the plaque (thick arrow) and pituitary stalk (thin arrow). **(E)** Axial view after contrast enhancement. **(F)** Sagittal view perpendicular to blood flow after contrast enhancement. **(G)** Coronal view after enhancement. The plaque (thick arrow) demonstrates minimal enhancement, lower than that of the pituitary stalk (thin arrow), and similar to the signal intensity of normal intracranial arterial vessel walls.

**Figure 2:**
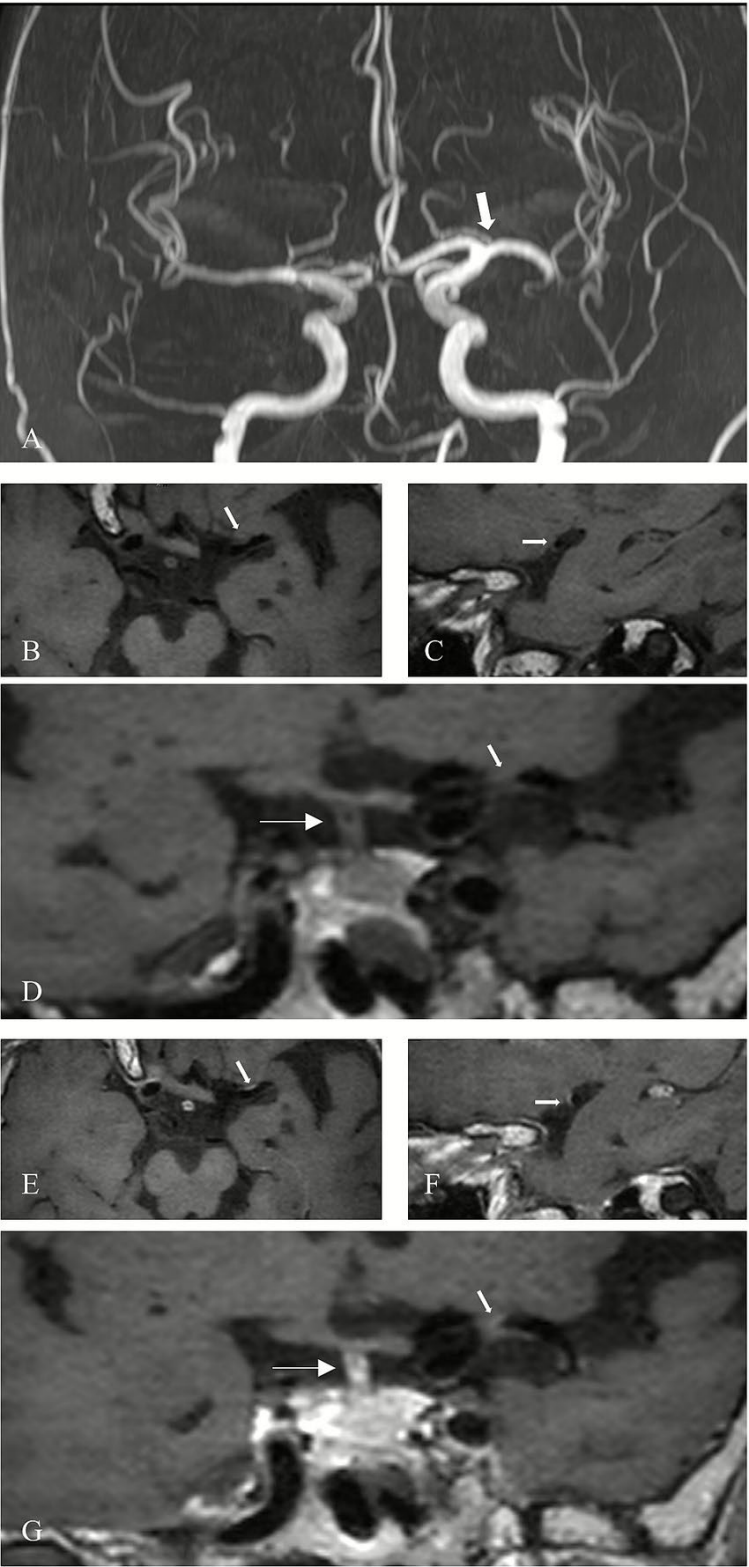
Grade I enhancement of a possible culprit plaque. **(A)** MRA shows mild stenosis of the left MCA (M1 segment) in a patient (arrow). **(B)** Axial 3D-MATRIX T1WI shows corresponding arterial wall thickening (arrow). **(C)** Sagittal reconstruction perpendicular to the direction of blood flow shows the eccentric plaque (arrow). **(D)** Coronal image shows the plaque (thick arrow) and pituitary stalk (thin arrow). **(E)** Axial view after contrast enhancement. **(F)** Sagittal view perpendicular to blood flow after contrast enhancement. **(G)** Coronal view after enhancement. The plaque (thick arrow) exhibits enhancement greater than normal intracranial arteries, but less than the pituitary stalk (thin arrow).

**Figure 3:**
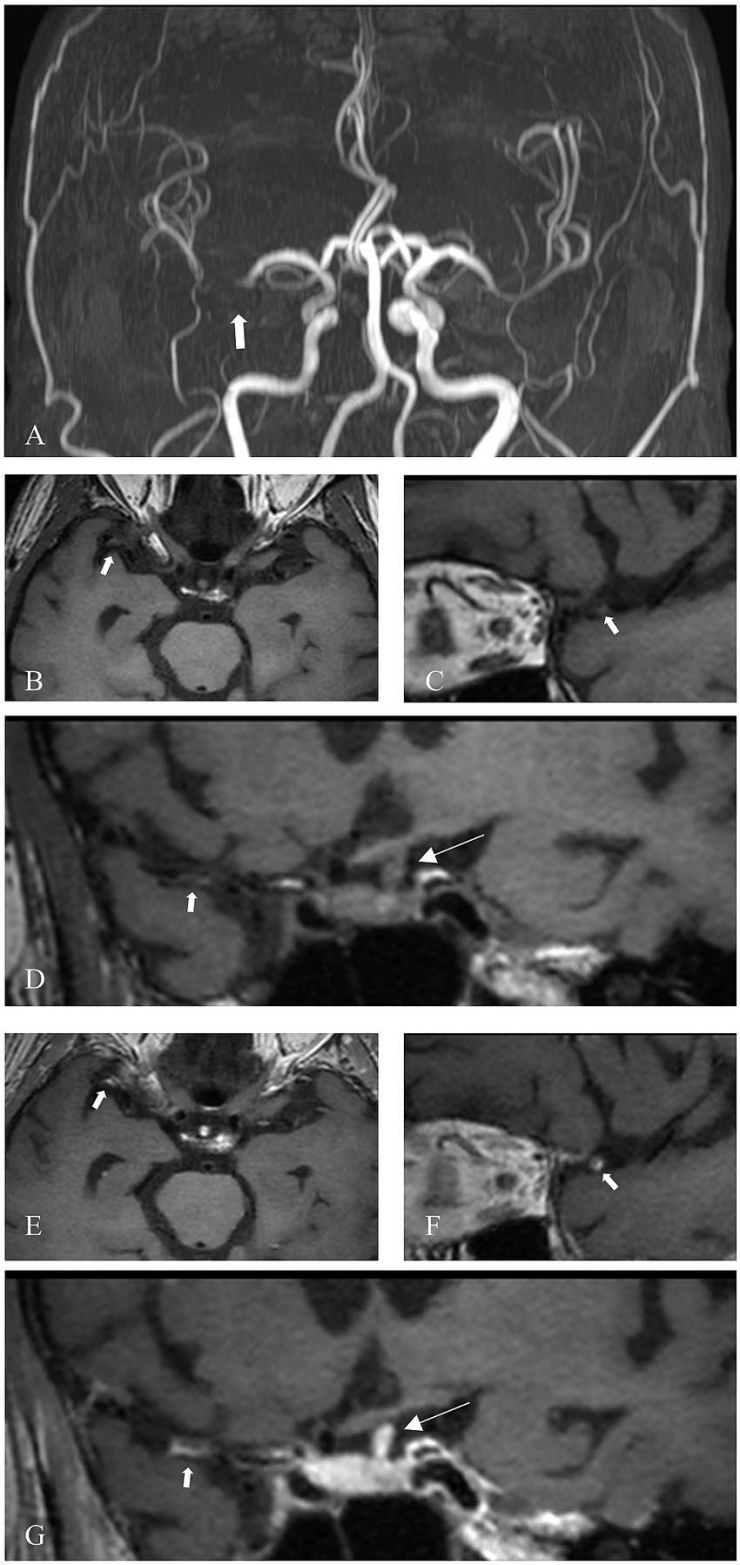
Grade II enhancement of a culprit plaque. **(A)** MRA shows severe stenosis of the right MCA (M1 segment) in a patient (arrow). **(B)** Axial 3D-MATRIX T1WI shows the corresponding plaque (arrow). **(C)** Sagittal reconstruction perpendicular to blood flow localizes the eccentric plaque (arrow). **(D)** Coronal image shows the plaque (thick arrow) and pituitary stalk (thin arrow). **(E)** Axial view after contrast enhancement. **(F)** Sagittal view perpendicular to blood flow after contrast enhancement. **(G)** Coronal view after enhancement. The plaque (thick arrow) demonstrates a higher degree of enhancement than the pituitary stalk (thin arrow).

Evaluation of plaque characteristics included assessment of contrast enhancement grade and the presence of T1-weighted hyperintensity. This evaluation was independently conducted by two additional neuroradiologists with 12 and 2 years of experience, who were blinded to clinical history, symptoms, and findings from conventional MRI sequences. Pre-contrast images were used as references, and contrast enhancement on post-contrast HRMR-VWI was qualitatively graded based on signal intensity using a previously validated classification system (16). Plaques were classified into the following categories: Grade 0: enhancement less than or equal to that of the normal vessel wall (Figure 1); Grade I: enhancement greater than Grade 0 and more intense than other normal intracranial arterial walls, but less than that of the pituitary stalk (Figure 2); Grade II: enhancement equal to or exceeding that of the pituitary stalk (Figure 3). T1 hyperintensity was defined as high signal intensity on T1WI FS, with signal intensity ≥ 1.5 times that of adjacent brain parenchyma or muscle tissue (17). In cases of disagreement between the two evaluators, consensus was reached through discussion under the supervision of a senior neuroradiologist with 14 years of experience.

### 2.4 Statistical analysis

All statistical analyses were performed using IBM SPSS Statistics version 26 (IBM Corp., Armonk, NY, USA). Categorical variables were presented as frequencies, and continuous variables were expressed as means ± standard deviation. The chi-squared (*χ*^2^) test was used to analyze differences in the incidence of T1WI hyperintensity among culprit, possible culprit, and non-culprit plaque groups. The Kruskal-Wallis H test was used to analyze differences in plaque contrast enhancement grades across the three groups. Multivariable logistic regression analysis was performed to assess the association between plaque characteristics—specifically contrast enhancement grade and T1WI hyperintensity— and the presence of culprit plaques. The Kappa coefficient was used to estimate the consistency of the evaluators in the classification of plaques, plaque enhancement, and T1 high signal assessment. A *κ* value less than 0.40 indicates poor consistency, a *κ* value of 0.40–0.60 is moderate, and a κ value greater than 0.60 is excellent. A *p*-value < 0.05 was considered statistically significant.

## 3 Results

### 3.1 Patients characteristics

**Figure 4:**
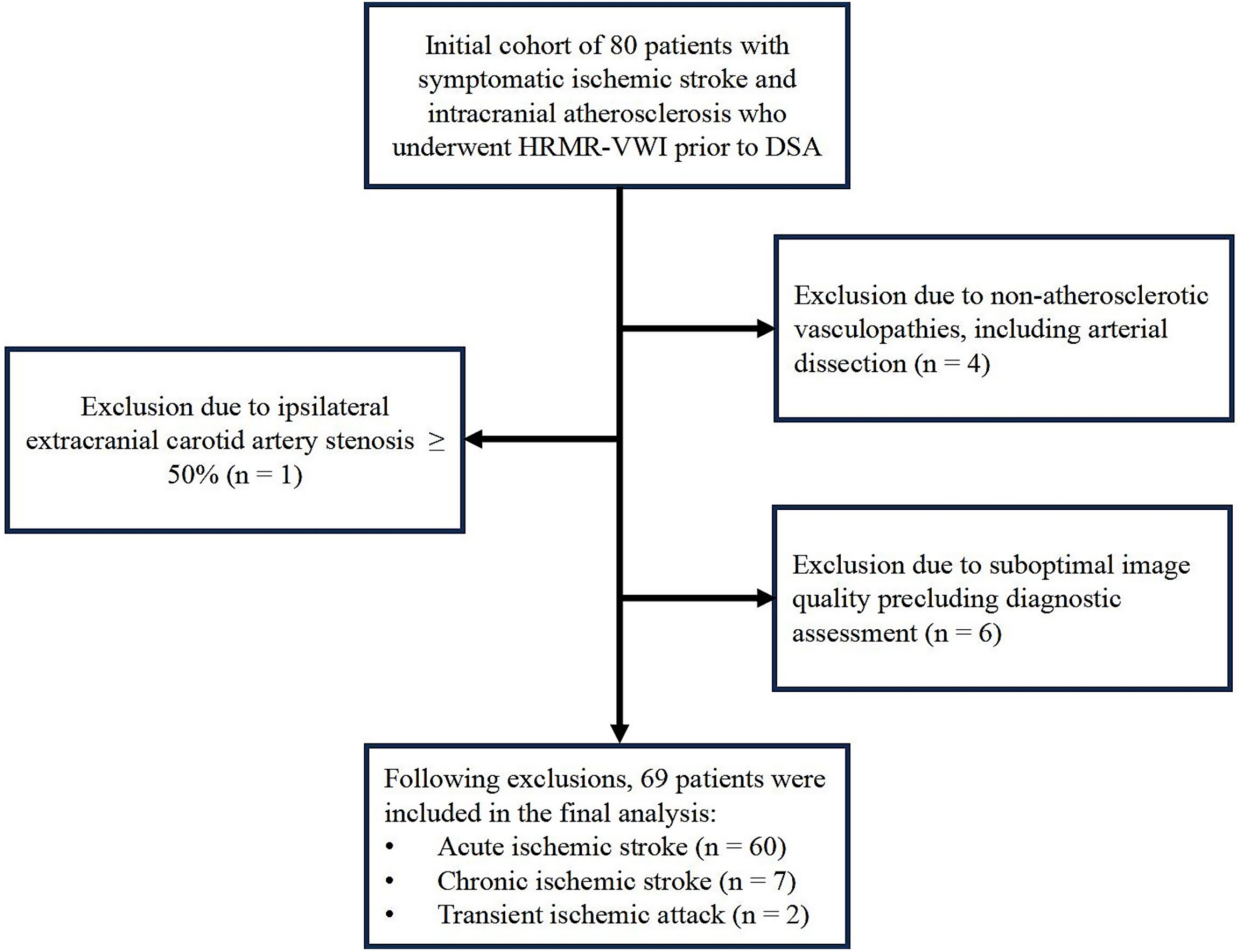
Flowchart of patient selection.

A total of 80 patients presenting with symptoms of ischemic stroke were initially screened at the Department of Neurology, Shanghai East Hospital (South Division). Intracranial artery stenosis was identified through computed tomography angiography and/or MRA, and was subsequently confirmed by DSA. All participants underwent HRMR-VWI of the intracranial arteries. Following the application of exclusion criteria, four patients with arterial dissection, 1 patient with ≥ 50% stenosis of the contralateral extracranial carotid artery, and 6 patients with inadequate image quality were excluded from the study. A total of 69 patients (50 males and 19 females; mean age 61.57 ± 11.82 years) were included in the final analysis. The participant selection process is illustrated in Figure 4. Of the 69 patients included, 60 were diagnosed with acute ischemic stroke, 7 with chronic ischemic stroke, and 2 with transient ischemic attack. Clinical characteristics of the study population are summarized in Table 1.

**Table 1:** Demographic and clinical characteristics of the study population.

Clinical characteristics	Results	Sample (*n* = 69)
Age	61.57 ± 11.82	
Male	50	72.50%
Hypertension	51	73.91%
Diabetes	19	27.54%
Coronary artery disease	4	5.80%
Stroke	17	24.64%
Hyperlipidemia	2	0.03%
Smoking history	16	23.19%
Smoking history	31	44.93%
Acute ischemic stroke	60	86.96%
Chronic ischemic stroke	7	10.14%
Transient ischemic attack	2	2.90%

Results are presented as percentages, mean ± standard deviation.

### 3.2 Intracranial atherosclerotic plaque characteristics

A total of 474 intracranial atherosclerotic plaques were identified in the study cohort. Among these, 382 plaques (80.59%) were located in the anterior circulation, while 92 plaques (19.41%) were located in the posterior circulation. Based on clinical and imaging criteria, 72 plaques (15.19%) were classified as culprit plaques (with 3 patients presenting with 2 culprit plaques each), 132 plaques (27.85%) were classified as possible culprit plaques, and 270 plaques (56.96%) were classified as non-culprit plaques (Table 2).

**Table 2:** Distribution of intracranial atherosclerotic plaques by plaque classification.

Plaque characteristics	Frequency	Composition ratio
Anterior cerebral artery	47	9.92%
Middle cerebral artery	235	49.58%
Internal carotid artery	100	21.10%
Basilar artery	48	10.13%
Vertebral artery	15	3.16%
Posterior cerebral artery	29	6.12%
Total	474	100.00%
Culprit plaque	72	15.19%
Possible culprit plaque	132	27.85%
Non-culprit plaque	270	56.96%

#### 3.2.1 Plaque contrast enhancement grades

**Figure 5:**
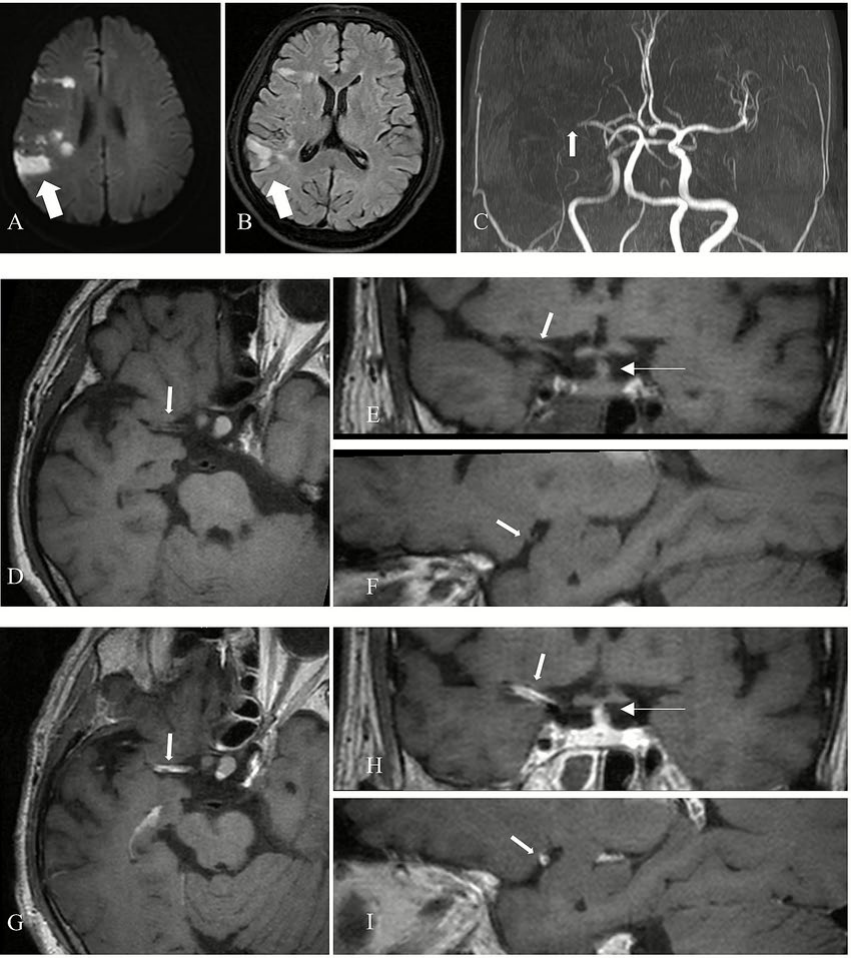
Grade II enhancement of a culprit plaque in a patient with acute cerebral infarction. **(A)** DWI shows acute cerebral infarction in the right frontal and parietal lobes (arrow). **(B)** FLAIR shows hyperintensity in the corresponding region (arrow). **(C)** MRA demonstrates severe stenosis in the right MCA (M1 segment) (arrow). **(D,G)** Axial pre- and post-enhancement contrast images identify the plaque in the right MCA (M1 segment) (arrow). **(E)** Coronal image shows the plaque (thick arrow) and the pituitary stalk (thin arrow). **(H)** Coronal post-contrast image shows the plaque (thick arrow) with a higher degree of enhancement than the pituitary stalk (thin arrow). **(F,I)** Sagittal reconstructions perpendicular to blood flow before and after contrast show the eccentric plaque (arrow).

Among the 72 culprit plaques, 53 plaques (73.61%) demonstrated grade II enhancement, 8 plaques (11.11%) showed grade I enhancement, and 11 plaques (15.28%) showed grade 0 enhancement. Among the possible culprit plaques (*n* = 132), 14 plaques (10.61%) showed grade II enhancement, 64 plaques (48.48%) showed grade I enhancement, and 54 plaques (40.91%) showed grade 0 enhancement. In the non-culprit group (*n* = 270), 20 plaques (7.41%) showed grade II enhancement, 67 plaques (24.81%) showed grade I enhancement, and 183 plaques (67.78%) showed grade 0 enhancement. The Kruskal-Wallis H test indicated a statistically significant difference in enhancement grades among the three plaque groups (*Z* = 116.310, *p* < 0.001). Pairwise comparisons between culprit and possible culprit plaques, as well as culprit and non-culprit plaques, showed statistically significant differences in contrast enhancement grades. Notably, the incidence of grade II enhancement was higher in culprit plaques compared with both possible culprit and non-culprit plaques (73.61% vs. 10.61% vs. 7.41%), as detailed in Table 3. An example of grade II enhancement in culprit plaques in acute ischemic stroke is shown in Figure 5.

**Table 3:** Kruskal-Wallis H test comparing contrast enhancement grades among culprit, possible culprit, and non-culprit plaques.

Items	II	I	0	*Z*	*p*
Culprit plaque	53 (73.61%)	8 (11.11%)	11 (15.28%)	116.310	<0.001
Possible culprit plaque	14 (10.61%)	64 (48.48%)	54 (40.91%)		
Non-culprit plaque	20 (7.41%)	67 (24.81%)	183 (67.78%)		

#### 3.2.2 T1WI hyperintensity

**Figure 6:**
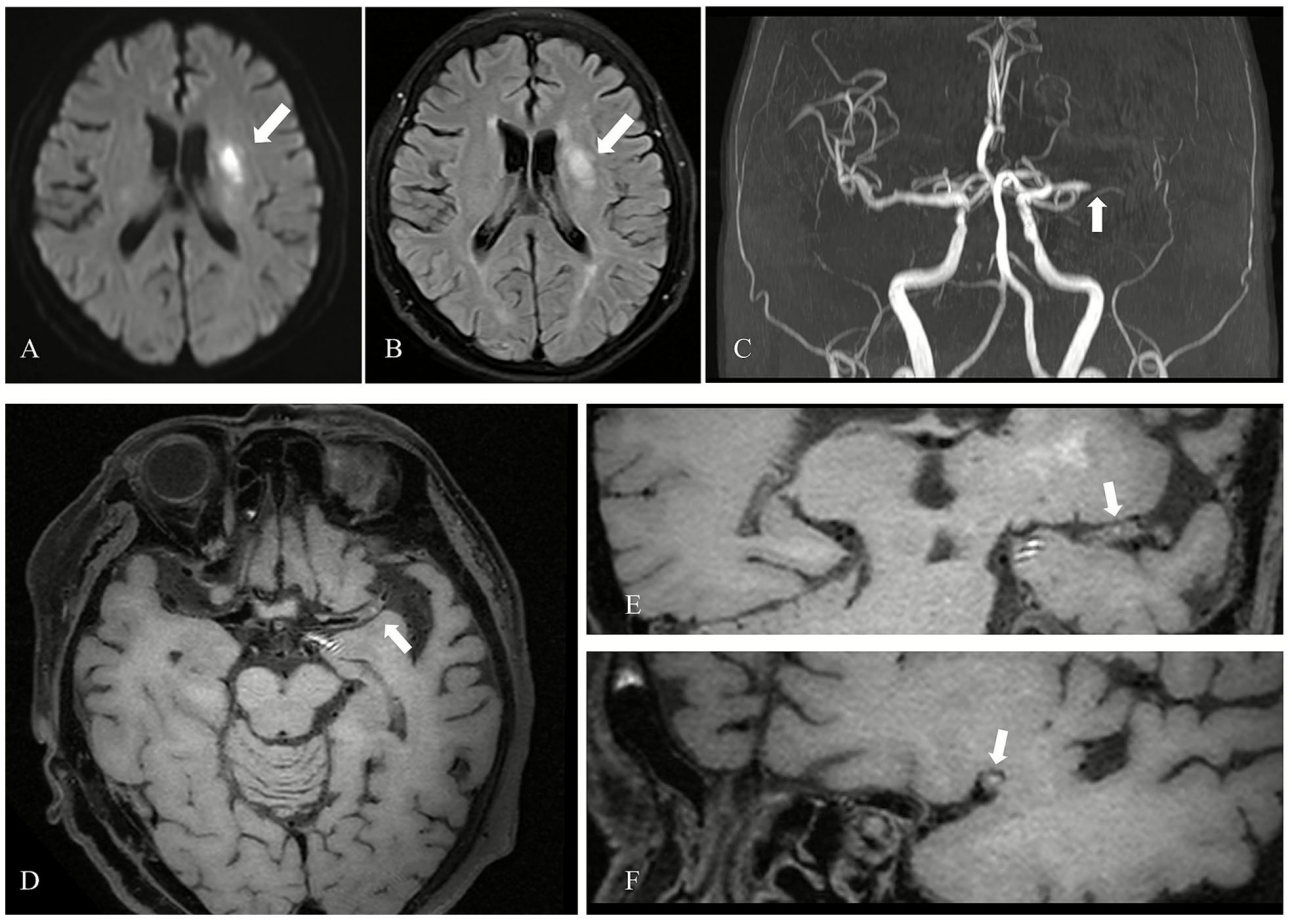
T1-weighted hyperintensity in a culprit plaque in a patient with acute cerebral infarction. **(A)** DWI shows an acute cerebral infarction in the left basal ganglia (arrow). **(B)** FLAIR shows hyperintensity at the corresponding region (arrow). **(C)** MRA shows occlusion in the left MCA (M1 segment) (arrow). **(D)** Axial 3D-MATRIX T1WI fat-suppressed shows a plaque with punctate hyperintensity (arrow), with signal intensity 1.5 times greater than that of adjacent brain tissue. **(E)** Coronal image reconstruction perpendicular to the flow direction confirm high signal within the plaque (arrow). **(F)** Sagittal image reconstruction perpendicular to the flow direction confirms high signal within the plaque (arrow).

Among the 72 culprit plaques, 25 plaques (34.72%) showed hyperintensity on the T1WI fat-saturated sequence, while 47 plaques (65.28%) did not. Among the 132 possible culprit plaques, 18 plaques (13.64%) showed hyperintensity on the T1WI fat-saturated sequence, while 114 plaques (86.36%) did not. Among the 270 non-culprit plaques, 5 plaques (1.85%) showed hyperintensity on the T1WI fat-saturated sequence, while 265 plaques (98.15%) did not. Statistical analysis using the *χ*^2^ test demonstrated a significant difference in the occurrence of T1WI hyperintensity among the three groups (*χ*^2^ = 69.958, *p* < 0.001). Pairwise comparisons between culprit and possible culprit plaques, as well as between culprit and non-culprit plaques, also showed statistically significant differences in the occurrence of T1 hyperintensity, as shown in Table 4. The incidence of T1WI hyperintensity was highest in culprit plaques compared with possible culprit and non-culprit plaques (34.72% vs. 13.64% vs. 1.85%). Representative T1WI hyperintensity in a culprit plaque from a patient with acute ischemic stroke is presented in Figure 6.

**Table 4:** *χ*^2^ test for the incidence of T1WI hyperintensity among culprit, possible culprit, and non-culprit plaques.

T1WI hyperintensity	Culprit plaque	Possible culprit plaque	Non-culprit plaque	*χ* ^2^	*p*
Yes	25 (34.72%)^a^	18 (13.64%)^b^	5 (1.85%)^c^	69.958	<0.001
No	47 (65.28%)	114 (86.36%)	265 (98.15%)		
Total	72 (100%)	132 (100%)	270 (100%)		

a,b,c Three groups were compared pairwise; shared letters indicate no significant difference between groups, while unshared letters indicate significant differences between groups.

### 3.3 Association between plaque features and culprit plaques

Multivariate logistic regression analysis was performed using non-culprit plaques as the reference group. Grade II enhancement was found to be independently and positively associated with culprit plaques when compared to grade 0 enhancement [odds ratio (OR) = 29.256, 95% confidence interval (CI) 12.519–68.371, *p* < 0.001]. In contrast, grade I enhancement did not demonstrate a significant association with culprit plaques compared to grade 0 enhancement (*p* = 0.169). Additionally, the presence of T1WI hyperintensity was independently and positively associated with culprit plaques [OR = 3.880, 95% Confidence intervals (95% CI) 1.262–11.928, *p* = 0.018]. Detailed results are presented in Table 5.

**Table 5:** Multivariate logistic regression analysis of plaque features associated with culprit plaques.

Plaque type	Item	OR	95%CI	*p*-value
Culprit plaque	T1 hyperintensity	3.880	(1.262, 11.928)	0.018
	T1 No hyperintensity (control)			
	Grade II enhancement	29.256	(12.519, 68.371)	*p* < 0.001
	Grade I enhancement	1.954	(0.753, 5.068)	0.169
	Grade 0 enhancement (control)			
Possible culprit plaque	T1 hyperintensity	10.573	(3.156, 35.417)	*p* < 0.001
	T1 No hyperintensity (control)			
	Grade II enhancement	0.898	(0.334, 2.415)	0.830
	Grade I enhancement	3.070	(1.930, 4.883)	*p* < 0.001
	Grade 0 enhancement (control)			

Reference category: non-culprit plaques.

### 3.4 Repeatability of MRI image measurements

The consistency in determining the lesion type (culprit plaque, possible culprit plaque, or non-culprit plaque) was very good (*κ* = 0.897, 95%CI: 0.859–0.931). In addition, the consistency in evaluating the plaque enhancement grade (grade II enhancement, grade I enhancement, or grade 0 enhancement; weighted *κ* = 0.821, 95% CI: 0.782–0.853) and the consistency in assessing whether the plaque had T1 high signal (*κ* = 0.797, 95% CI: 0.699–0.878) was excellent.

## 4 Discussion

This study demonstrated that grade II contrast enhancement and T1WI hyperintensity of intracranial atherosclerotic plaques were independently and positively associated with culprit plaques in patients with symptomatic intracranial atherosclerosis. Significant differences in contrast enhancement grade and the prevalence of T1WI hyperintensity were observed among culprit, possible culprit, and non-culprit plaques. Culprit plaques more frequently exhibited Grade II enhancement and T1WI hyperintensity, while non-culprit plaques were predominantly characterized by grade 0 enhancement and absence of hyperintensity. These findings may serve as markers of plaque instability and are potentially useful in distinguishing high-risk plaques associated with ischemic stroke and contribute to further risk stratification. At present, there is a lack of Class I evidence from large-scale randomized controlled trials to formulate specific treatment decisions or follow-up plans based on the imaging features of HR-VWI. Therefore, our findings should be regarded as providing important pathophysiological basis and prospective hypotheses for the future construction of individualized secondary prevention strategies.

Plaque enhancement and IPH are widely recognized imaging features of unstable atherosclerotic plaques and may serve as predictors of plaque progression and stroke recurrence. Plaque rupture and *in situ* thrombosis may lead to artery-to-artery embolism or arterial occlusion, which are considered principal mechanisms underlying stroke associated with intracranial atherosclerotic stenosis (18). Histopathological studies of carotid artery plaques have shown increased macrophage infiltration and neovascularization in ruptured plaques, supporting the central role of inflammation in plaque destabilization (19). HRMR-VWI studies of carotid plaques have further supported this association, indicating that plaque enhancement correlates with neovascularization, and macrophage infiltration, indirectly reflecting the inflammatory activity of the plaque (20). Additional HRMR-VWI research has demonstrated that plaque enhancement is associated with neovascularization, active inflammation, and contrast agent leakage caused by endothelial dysfunction. These features not only serve as indirect markers of inflammation but also independently correlate with recent cerebrovascular ischemic events and stroke recurrence (21–23). Despite these findings, the pathological mechanisms underlying plaque enhancement in intracranial arteries remain incompletely understood. An early study by Amarenco et al. (2006) reported no significant association between intracranial plaque enhancement and ischemic stroke events (ISEs) (24). However, the reliability of this conclusion is limited by a small sample size (*n* = 23). In recent years, research has increasingly demonstrated a significant positive association between the degree of intracranial plaque enhancement and the occurrence of ISEs. Multiple clinical studies have confirmed a significant positive correlation between plaque enhancement and the incidence of ISEs (16, 25–27). Nevertheless, several methodological limitations are prevalent in the existing literature. First, more than 50% of studies have included cases with arterial stenosis > 50%, even though severe stenosis itself is a well-established contributor to acute ISEs. Second, many of these studies have not simultaneously analyzed the relationship between arterial stenosis severity and ISEs, making it difficult to distinguish the independent effects of plaque enhancement from those of luminal narrowing. This limitation is also present in the current study, which did not adjust for additional vulnerable plaque features—such as the degree of stenosis, plaque thickness, and plaque burden—all of which are known risk factors for ISEs. Thus, the potential for confounding cannot be excluded.

In addition to its diagnostic value, plaque enhancement may serve as an imaging biomarker for evaluating treatment response. Chung et al. (28) reported a significant reduction in enhancement volume among 77 patients treated with statin therapy. A prospective study using 7 T HR-MRI by Sanchez et al. (29) demonstrated a strong correlation between reduced enhancement and decreased low-density lipoprotein cholesterol levels (*r* = 0.8). These findings support the potential utility of plaque enhancement as a non-invasive imaging marker for assessing treatment efficacy and monitoring disease progression in patients with intracranial atherosclerosis.

Histopathological studies of carotid arteries have indicated T1-weighted hyperintensity is a reliable marker of recent IPH (30). IPH is caused by the rupture of fragile microvessels and neovessels within the plaque, and is closely associated with plaque progression, fibrous cap thinning or rupture, and the presence of neurological symptoms (31). A meta-analysis extracranial carotid artery plaques conformed T1 hyperintensity as a significant predictor of subsequent stroke or transient ischemic attack (32). Furthermore, studies have reported significantly higher incidence of T1 hyperintensity in symptomatic MCA plaques compared to asymptomatic plaques (33, 34). A recent systematic review and meta-analysis of HRMR-VWI studies found that T1 hyperintensity is more frequently observed in symptomatic atherosclerotic stenosis than in asymptomatic cases, suggesting that ICAS may share common underlying pathophysiological mechanisms with carotid atherosclerosis (35).

However, anatomical differences between extracranial and intracranial arteries pose challenges in detecting and interpreting IPH. Intracranial arteries are smaller in caliber and located in deeper anatomical regions, which may contribute to a lower reported prevalence of IPH. Postmortem studies have shown that IPH occurs less frequently in intracranial plaques compared to their extracranial counterparts. In all existing HRMR-VWI studies of symptomatic MCA plaques, the incidence of IPH remains below $\frac{1}{3}$ . Moreover, it is important to recognize that T1 hyperintensity on MRI is not specific to hemorrhage; it may also reflect the presence of fat, metal deposition, or high protein concentration. Previous studies have recommended the combined use of fat-suppressed T1-weighted imaging and TOF sequences for IPH detection, which together provide high sensitivity and specificity (30).

In the present study, the combination of T1-weighted fat-suppressed imaging and TOF MRA was used to identify T1 hyperintensity. Among 72 culprit plaques, 25 (34.72%) exhibited T1 hyperintensity. Multivariate logistic regression analysis confirmed a significant association between T1 hyperintensity and culprit plaques, consistent with previous research. However, unlike the carotid artery, the intracranial arteries are not readily accessible for tissue sampling, and histopathological validation of HRMR-VWI findings in intracranial IPH remains limited. The diagnostic criteria for IPH used in this study—as well as in existing literature—are derived from carotid artery studies and postmortem histopathological comparisons. Thus, the correlation between HRMR-VWI characteristics and the histopathology of intracranial IPH has not yet been validated using a definitive gold standard.

An interesting observation in the present study was that a subset of culprit plaques exhibited grade 0 enhancement. This finding has been reported in previous studies. In a study by Lu et al. (36), among 44 culprit plaques identified in patients with acute or subacute stroke, 1 plaque (2.3%) showed no enhancement (grade 0). Similarly, Cheng et al. (15) reported that 7 out of 231 culprit plaques exhibited grade 0 enhancement. However, in an earlier study involving 20 patients with acute ischemic stroke (onset < 4 weeks) reported that all 21 identified culprit plaques demonstrated contrast enhancement, with none classified as grade 0 (16). This discrepancy may be attributed to differences in sample size or differences in the timing of imaging relative to the ischemic event. A study by Skarpathiotakis et al. (37) reported that within the first 4 weeks of an ischemic stroke, marked enhancement of intracranial atherosclerotic plaques was consistently observed in plaques within the infarct territory. However, the degree of enhancement diminished progressively with time elapsed since the ISE. The current study included patients with a range of ischemic presentations, including acute stroke, chronic stroke, and transient ischemic attacks. Notably, both grade II enhancement and T1WI hyperintensity were independently associated with culprit plaques. These findings support the utility of HRMR-VWI features in identifying high-risk intracranial plaques and may aid in predicting the likelihood of future ischemic events.

This study has several limitations. First, it was a single-center, retrospective analysis with a limited sample size. This may affect the statistical power of our research results. Specifically, it limits our ability to conduct meaningful subgroup analyses and to construct complex multivariate models that incorporate more variables. Given the low prevalence of symptomatic ICAS and the difficulty in completing HRMR-VWI scans, in the initial study design, we aimed to explore the general value of HRMR-VWI features in identifying “culprit plaques,” and thus included all assessable intracranial large artery plaques to increase statistical power and the generalizability of the results. Due to the anatomical and hemodynamic differences among the internal carotid artery, anterior circulation, and posterior circulation, the characteristics and stability of culprit plaques vary among different vessels, and direct comparisons of plaques in different vessels may indeed introduce heterogeneity. Future studies with larger sample sizes are needed to conduct in-depth analyses specifically targeting single vessel types (such as MCA or basilar artery) to reveal possible vessel-specific differences. As a retrospective study, the study is subject to inherent selection bias, with some patients potentially excluded due to severe clinical conditions or suboptimal image quality. Although significant associations were identified between certain imaging features and culprit plaques, large-scale, prospective, multicenter longitudinal studies are needed, which enables sufficient statistical power for conducting reliable subgroup analyses to further validate the diagnostic value of grade II enhancement and T1-weighted hyperintensity in accurately identifying culprit plaques. Thus, specific biomarkers in different patient groups can be identified. Through long-term follow-up, the predictive value of HR-VWI features (such as plaque enhancement and high T1 signal) for the risk of future stroke recurrence can be clearly verified, which is the ultimate goal of achieving individualized secondary prevention. Second, the analysis was performed at the plaque level rather than at the individual level, and the study did not account for the potential influence of vascular risk factors on plaque characteristics. Recent evidence indicates that variables such as age, hypertension, and diabetes are positively correlated with the number of intracranial vessel wall lesions (38). This study mainly focused on the assessment of plaque vulnerability by comparing enhancement and high T1 signal. However, a comprehensive evaluation should integrate multiple sequence information and various morphological parameters to more accurately reflect the vulnerability of the plaque. For instance, T2-weighted imaging is crucial for identifying lipid necrotic cores and assessing the integrity of the fibrous cap; while morphological parameters such as positive remodeling, plaque burden, stenosis degree, eccentricity index, and involvement of perforating arteries are also indispensable elements for judging plaque stability and clinical risk. Future case–control studies are warranted to explore threshold values for plaque enhancement, refine definitions of plaque vulnerability—including parameters such as positive remodeling, plaque burden, degree of stenosis, eccentricity index, and involvement of perforating arteries. Comprehensive studies based on multi-parameter MRI will help to establish a more accurate risk prediction model for intracranial atherosclerotic plaques. Third, interpretation of plaque imaging features in this study was largely extrapolated from carotid artery research. Due to the significant ethical and practical difficulties in obtaining histological specimens of intracranial arteries from living individuals, the HR-VWI characteristics of intracranial plaques currently lack the gold standard of histopathology for verification. Therefore, the exact pathological basis (such as intraplaque hemorrhage, lipid-rich necrotic core, or inflammatory cell aggregation) of the high signal of intracranial artery plaques on T1-weighted images is still not fully clear, which might the result of the combined action of multiple components. Therefore, when interpreting our results, it should be cautious and not simply and directly equate the T1 high signal with definite intraplaque hemorrhage. It should rather be regarded as a high-risk imaging biomarker representing plaque instability. Given the anatomical and physiological differences between extracranial and intracranial arteries, and the current lack of pathological validation for HRMR-VWI findings in intracranial plaques, further research is necessary to establish correlations between imaging features and histopathological evidence specific to intracranial atherosclerosis.

## 5 Conclusion

In patients with symptomatic ICAS, grade II plaque enhancement and T1WI hyperintensity were independently associated with culprit plaques and demonstrated strong discriminatory value in their identification. These imaging features may serve as noninvasive markers of plaque vulnerability, contributing to increases risk stratification and enabling earlier preventive interventions aimed at reducing the likelihood of recurrent ischemic events.

## Data Availability

The raw data supporting the conclusions of this article will be made available by the authors, without undue reservation.
